# Characterizing hub biomarkers for post-transplant renal fibrosis and unveiling their immunological functions through RNA sequencing and advanced machine learning techniques

**DOI:** 10.1186/s12967-024-04971-9

**Published:** 2024-02-20

**Authors:** Xinhao Niu, Cuidi Xu, Yin Celeste Cheuk, Xiaoqing Xu, Lifei Liang, Pingbao Zhang, Ruiming Rong

**Affiliations:** 1 Department of Urology, Zhongshan Hospital, Fudan University, Shanghai, 200032 China; 2 Shanghai Key Laboratory of Organ Transplantation, Shanghai, 200032 China; 3 Department of Urology, Huashan Hospital, Fudan University, Shanghai, 200040 China

**Keywords:** Kidney transplant, Renal fibrosis, Immune microenvironment, Biomarkers, WGCNA

## Abstract

**Background:**

Kidney transplantation stands out as the most effective renal replacement therapy for patients grappling with end-stage renal disease. However, post-transplant renal fibrosis is a prevalent and irreversible consequence, imposing a substantial clinical burden. Unfortunately, the clinical landscape remains devoid of reliable biological markers for diagnosing post-transplant renal interstitial fibrosis.

**Methods:**

We obtained transcriptome and single-cell sequencing datasets of patients with renal fibrosis from NCBI Gene Expression Omnibus (GEO). Subsequently, we employed Weighted Gene Co-Expression Network Analysis (WGCNA) to identify potential genes by integrating core modules and differential genes. Functional enrichment analysis was conducted to unveil the involvement of potential pathways. To identify key biomarkers for renal fibrosis, we utilized logistic analysis, a LASSO-based tenfold cross-validation approach, and gene topological analysis within Cytoscape. Furthermore, histological staining, Western blotting (WB), and quantitative PCR (qPCR) experiments were performed in a murine model of renal fibrosis to verify the identified hub genes. Moreover, molecular docking and molecular dynamics simulations were conducted to explore possible effective drugs.

**Results:**

Through WGCNA, the intersection of core modules and differential genes yielded a compendium of 92 potential genes. Logistic analysis, LASSO-based tenfold cross-validation, and gene topological analysis within Cytoscape identified four core genes (CD3G, CORO1A, FCGR2A, and GZMH) associated with renal fibrosis. The expression of these core genes was confirmed through single-cell data analysis and validated using various machine learning methods. Wet experiments also verified the upregulation of these core genes in the murine model of renal fibrosis. A positive correlation was observed between the core genes and immune cells, suggesting their potential role in bolstering immune system activity. Moreover, four potentially effective small molecules (ZINC000003830276-Tessalon, ZINC000003944422-Norvir, ZINC000008214629-Nonoxynol-9, and ZINC000085537014-Cobicistat) were identified through molecular docking and molecular dynamics simulations.

**Conclusion:**

Four potential hub biomarkers most associated with post-transplant renal fibrosis, as well as four potentially effective small molecules, were identified, providing valuable insights for studying the molecular mechanisms underlying post-transplant renal fibrosis and exploring new targets.

**Supplementary Information:**

The online version contains supplementary material available at 10.1186/s12967-024-04971-9.

## Introduction

Kidney transplantation represents the most effective renal replacement therapy for patients facing end-stage renal disease. Advancements in matching techniques, the continuous evolution of immunosuppressive agents, and a deepened understanding of the pathophysiological mechanisms behind transplant kidney rejection [1] have led to impressive one-year and ten-year transplant kidney survival rates exceeding 90% and 54.3%, respectively [2]. However, post-transplant renal fibrosis poses a notable clinical challenge. Immunological mechanisms affect chronic allograft injury (CAI) induced fibrosis and allograft survival via different mechanistic signaling pathways [3], and alloantigen mediated cytotoxic T lymphocytes play an important role in chronic allograft injury and renal fibrosis [4]. In addition, the innate immune response induced by ischemia–reperfusion injury is also a factor in allografts injury and fibrosis after renal transplantation [5]. Histologically, post-transplant renal interstitial fibrosis is characterized by an increase in extracellular collagen fibers, tubular atrophy, and damage to peritubular capillaries [6]. This fibrotic transformation represents a common pathway to irreversible kidney damage, influenced by various harmful factors. Prior research has indicated that reduced peritubular capillary density resulting from kidney fibrosis leads to decreased renal perfusion. Consequently, this diminishes tissue oxygen availability, fosters the infiltration of inflammatory cells, and accelerates the generation of oxygen free radicals, all contributing to tissue fibrosis progression [7]. Interstitial fibrosis [8], tissue oxygenation status [9], and peritubular capillary integrity [10] are pivotal factors influencing the advancement of transplant kidney damage and the overall survival of the transplanted kidney.

Presently, the evaluation of renal interstitial fibrosis extent in transplant kidneys necessitates invasive kidney biopsies for a definitive diagnosis. However, this procedure entails risks such as graft bleeding, peri-renal infections, and potential patient fatalities. Notably, a reliable biological marker for diagnosing post-transplant renal interstitial fibrosis is lacking in clinical practice. With the advancement of sequencing technologies, analyzing disease biomarkers has emerged as a viable alternative.

In this study, utilizing transcriptome and single-cell sequencing data from databases containing patients with kidney fibrosis, our objective was to identify central biomarkers for renal fibrosis. Through logistic analysis, tenfold cross-validation with LASSO, and gene topological analysis in Cytoscape, we pinpointed four core genes significantly associated with renal fibrosis: CD3G, CORO1A, FCGR2A, and GZMH. These four genes demonstrated outstanding predictive performance in the validation set. Experimental validation through Western blotting (WB) and quantitative PCR (qPCR) confirmed the elevated expression of these core genes in a murine model of renal fibrosis. Furthermore, all core genes exhibited a positive correlation with core immune cells, suggesting their potential role in enhancing immune system activity in renal fibrosis and consequently contributing to disease progression.

## Materials and methods

### Datasets and data pre-processing

RNA-seq datasets, including GSE22459, GSE76882, GSE135327, and GSE65326 were downloaded from the GEO database. The datasets GSE22459, GSE76882, and GSE135327 were subjected to log normalization, and the batch effects were removed using the “combat” function from the sva package [11]. These processed datasets were defined as the screening set, consisting of 100 samples with kidney fibrosis and 136 normal samples. The same method was applied to validate the results using the validation set GSE65326. For single-cell data analysis, with the dataset sourced from GSE183837, including three Ctrl samples and six RIF samples.

### Screening of potential hub biomarkers

In the WGCNA analysis [12], the top 25% of genes with the highest variance were selected as the input matrix from the screening set. No samples were excluded. Topological calculations were performed using a range of soft-thresholding powers from 1 to 20. Based on the optimal soft-thresholding power (β = 11), the correlation matrix was transformed into an adjacency matrix and then converted into a Topological Overlap Matrix (TOM). Average linkage hierarchical clustering was performed using TOM to classify related modules, with each module containing at least 30 genes. Subsequently, similar modules were merged based on a cut height of 0.25. The correlation between the merged modules and the occurrence of kidney fibrosis was calculated using the Pearson method, and the module with the highest correlation was identified as the core module.Additionally, differential expression genes (DEGs) were selected from the screening set using the limma package [13]. DEGs were identified using the criteria of |log2 fold change (FC)|> 1 and adjusted p-value (adj.P.value)  < 0.05. The core genes within the core module were then overlapped with the DEGs to identify potential hub genes.

### Screening of hub biomarkers using machine learning

Logistic analysis was performed to determine the odds ratios (OR) of potential hub genes and understand their contributions to the development of kidney fibrosis. Redundant genes were eliminated using tenfold cross-validation in the LASSO regression (glmnet package [14]). The Support Vector Machine-Recursive Feature Elimination (SVM-RFE) method [15, 16] was then applied to further select genes. The overlapping genes obtained from the LASSO regression and SVM-RFE were identified as the final set of genes.

Subsequently, the identified genes were input into the STRING database to construct a protein–protein interaction (PPI) network. Various algorithms in Cytoscape were used to calculate the topological features of each gene. The maximum overlap of different methods was used to determine the core genes.

Finally, the diagnostic performance of the combined set of core genes for kidney fibrosis was validated using multiple machine learning methods: Bayes, Decision Tree (DT), Fisher Discriminant Analysis (FDA), Gradient Boosting Machine (GBM), Neural Networks (NNET), Random Forest (RG), and XGBoost.

### Enrichment analysis

GO enrichment analysis is a commonly used bioinformatics method that explores comprehensive information about large-scale gene data, including Biological Process (BP), Cellular Component (CC), and Molecular Function (MF) categories. Additionally, KEGG pathway enrichment analysis is widely used to understand biological mechanisms and functions. Meanwhile, Disease Ontology (DO) enrichment analysis can investigate the diseases primarily associated with the relevant genes. Finally, the results of GO, KEGG pathway, and DO analyses can be visualized using the GOplot package [17].

To further explore important signaling pathways related to the core genes, the clusterProfiler package [18] and GSVA package [19] can be utilized.

### Construction of regulatory network

Firstly, the mirDIP database [20] can be used to predict potential miRNAs targeting hub genes and identify the regulatory network of miRNAs. The upstream regulatory network can be established by selecting TF-core gene interactions with a p-value < 0.05 from the TRRUST database [21]. Additionally, the Comparative Toxicogenomics database [22] can be queried to identify compounds potentially associated with the core genes. Finally, the regulatory network of core genes can be visualized using the NetworkAnalyst database [23].

### Immune-related algorithm

The MCPcounter algorithm calculates the proportions of different immune cell types based on the expression levels of immune cell-related genes. The ssGSEA algorithm evaluates the activity of different immune functions. Finally, the outputs of infiltrating immune cells and immune functions are integrated to generate a matrix for analysis.

### Screening of hub immune cells

Differences in immune cell composition among different tissues are explored using the Wilcoxon test. Simultaneously, the Random Forest package is used to construct random forest trees for immune cells, determining the points with the lowest error. The immune cells are then ranked based on their importance, and genes with importance scores greater than 2 are selected. Ultimately, the overlapping immune cells identified above are screened to identify the core immune cells that may affect the occurrence of kidney fibrosis. Immunological analysis identifies two core immune cells that may influence the development of kidney fibrosis: Cytotoxic lymphocytes and T cells.

### Animal model

The murine renal ischemia–reperfusion injury models have been utilized in many studies to validate the results of bioinformatics analysis from transplant patients datasets [24]. In the light of the previous literature, we chose the murine IRI induced renal fibrosis model. Male C57BL/6 mice (6 weeks old, 20–25 g) were obtained from Shanghai JieSiJie Laboratory Animal Co., Ltd. (Shanghai, China) and housed in a specific pathogen-free (SPF)-grade animal facility. The mice were randomly assigned to two groups, and kidney samples from both groups were harvested at day 28 after surgery.

Sham Group: The abdomen was exposed for 30 min without clamping of the renal artery.

Ischemia–Reperfusion Injury (IRI) Group: Vascular clamps were applied to the renal pedicles of unilateral kidneys for 30 min to induce ischemia.

All animal procedures were conducted in compliance with the ethical guidelines and approved by the Animal Ethics Committee of Zhongshan Hospital, Fudan University.

### Quantitative PCR (qPCR)

Total RNA was extracted from each sample using TRIzol reagent (Thermo, CA, USA). Approximately 1000 ng of total RNA was reverse-transcribed into cDNA using the PrimeScript™ RT reagent kit (TaKaRa, Japan). mRNA levels were quantified using SYBR Green-based quantitative real-time PCR on an Applied Biosystems Real-time PCR System. All primers used in the study were synthesized by Sangon Biotech (Shanghai, China). The mRNA expression levels were normalized to GAPDH for comparative analysis. The detailed primer sequences are shown in Additional file 2: Table S1.

### Western blot analysis

Proteins were separated using 10% polyacrylamide denaturing gels and subsequently transferred onto polyvinylidene fluoride membranes by electroblotting. The primary antibodies employed included CORO1A (1:1000, TP71761, Abmart, China), CD3G (1:1000, T58478, Abmart, China), FCGR2A (1:1000, PHZ8459, Abmart, China), and GZMH (1:1000, PA1522, Abmart, China). The results were standardized against either GADPH (1:1000 dilution, M20024, Abmart, China) or β-Actin (1:1000 dilution, T40104, Abmart, China).

### Histological analysis

For morphological assessments, renal tissues preserved in paraffin, 28 days post-IRI, were sectioned into 5 μm slices. Subsequently, these sections were deparaffinized, rehydrated, and subjected to staining with hematoxylin and eosin (H&E), Sirius red, and Masson trichrome.

### Immunohistochemistry

Immunohistochemistry was conducted on thin Sects. (5 μm) of formalin-fixed and paraffin-embedded tissues. The sections underwent deparaffinization in xylene and rehydration using a descending series of ethanol. Tissue antigens were repaired by microwave heating, followed by incubation with 10% normal goat serum to block nonspecific reactions at room temperature for 10 min. Monoclonal rabbit primary antibodies against CD3G (1:200; Abcam, UK), CORO1A (1:200; Abcam, UK), FCGR2A (1:200; Abcam, UK), and GZMH (1:200; Abcam, UK) were applied separately and incubated overnight at 4 °C. Biotin-labeled goat anti-mouse/rabbit IgG and streptavidin-peroxidase (UltraSensitiveTM SP IHC Kit; Maxim, China) were subsequently used. The sections were then developed using diaminobenzidine substrate.

### Molecular docking and molecular dynamics simulations

The protein structure was retrieved from UniProt, screened as human species in the database, the full-length AlphaFold predicted structure was selected as the protein structure file, and the structure files of all FDA-approved drug small molecules were downloaded from the ZINC database, and virtual screening was performed using the Dock module in MOE v2022.02. The binding regions were predicted by SiteFinder, and the receptor proteins were set as rigid for virtual screening, and the best binding scores were generated. Based on the binding scores, sorting was performed, and among all the docking results, duplicates were removed and the top 20 drug small molecules with strong binding effects were retained for docking using the induced fit docking method in the Dock module.

100 ns molecular dynamics (MD) simulations were performed using GROMACS 2020.6 software to further validate the reasonableness and reliability of the docking results. The OPLS-AA/L all-atom force field and Amber GAFF force field were utilized to generate parameter and topology files for proteins and small molecule ligands, respectively. Periodic boundary conditions were set and optimized to simulate the size of the limiting box to fill the box with water molecules. To make the simulated system electrically neutral, some of the solvent water molecules were replaced with Na + and Cl- at a concentration of 0.15 mol/L. Use of the steepest descent method to minimize the energy consumption of the whole system. Pre-equilibrium was performed in two phases, the first phase equilibrium was simulated using the NVT system at 300 K and 100 ps to stabilize the temperature of the system, and the second phase equilibrium was simulated using the NPT system at 1 bar and 100 ps to stabilize the pressure of the system.

### Statistical analysis

All statistical analyses were performed with GraphPad Prism software version 8.0 (GraphPad.

Software, San Diego, CA, USA). Differences between experimental groups were assessed by one-way analysis of variance (ANOVA). Data are presented as the means ± SEMs. P values < 0.05 were considered statistically significant.

## Results

### WGCNA and differential expression analysis were used for screening

**Fig. 1:**
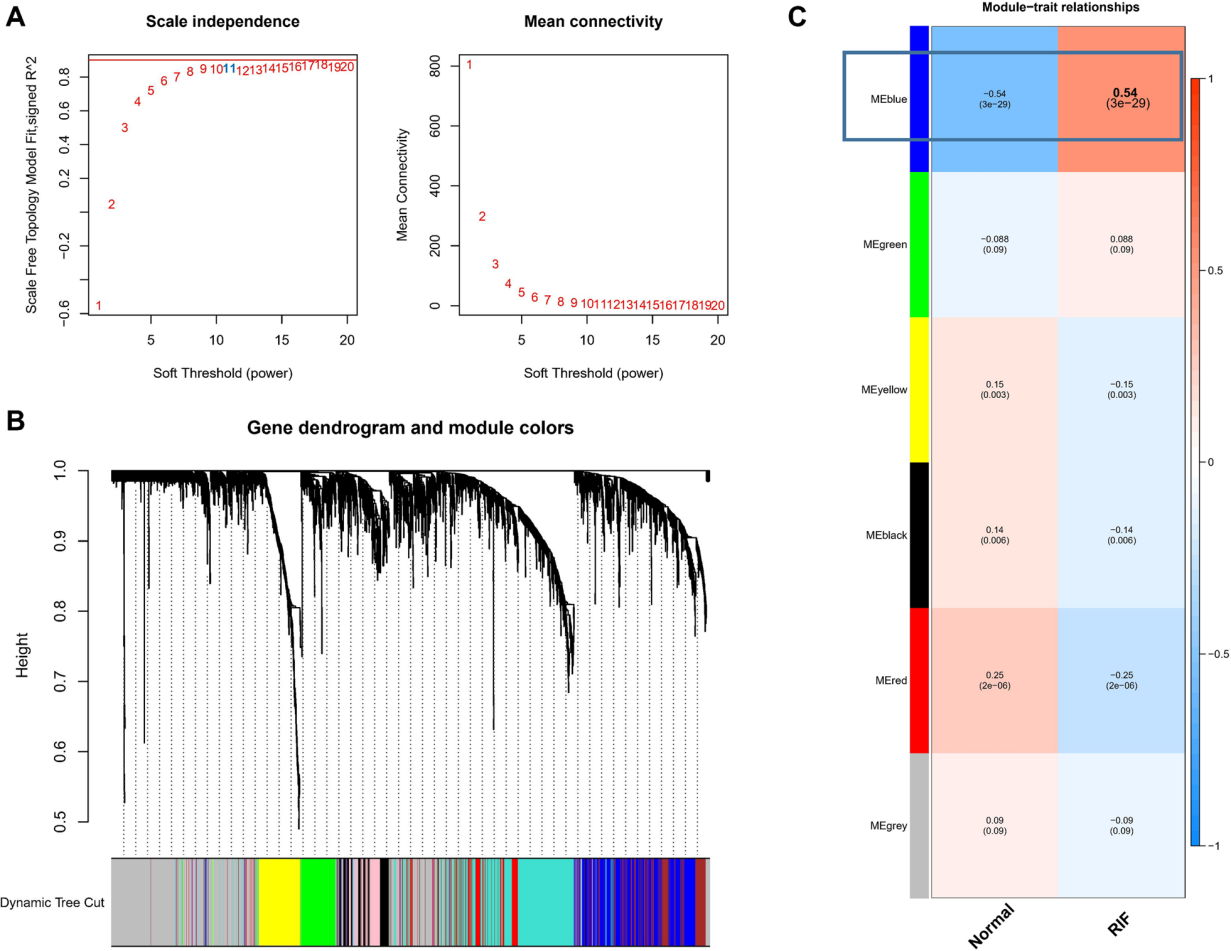
WGCNA analysis identified core modules associated with renal fibrosis. **A** Analysis of the scale-free fit index for various soft-thresholding powers (β) and the mean connectivity for various soft-thresholding powers. **B** The dendrogram of all genes is clustered based on a dissimilarity measure (1-TOM). **C** The heatmap shows six modules were identified between Normal and RIF. Red and blue represents a positive/negative correlation between MEs and samples

To establish a link between clinical information and key genes, WGCNA analysis was conducted. The samples displayed robust clustering with no outliers. Topological calculations were executed with a soft-thresholding power ranging from 1 to 20, and the optimal soft-thresholding power of 11 was determined (Fig. 1a). Utilizing this soft-thresholding power, the correlation matrix underwent transformation into an adjacency matrix and was subsequently converted into a Topological Overlap Matrix (TOM). Average linkage hierarchical clustering, utilizing TOM, was applied to classify related gene modules, each containing a minimum of 30 genes (Fig. 1b). Merging similar gene modules resulted in the identification of six modules (Fig. 1c).

**Fig. 2:**
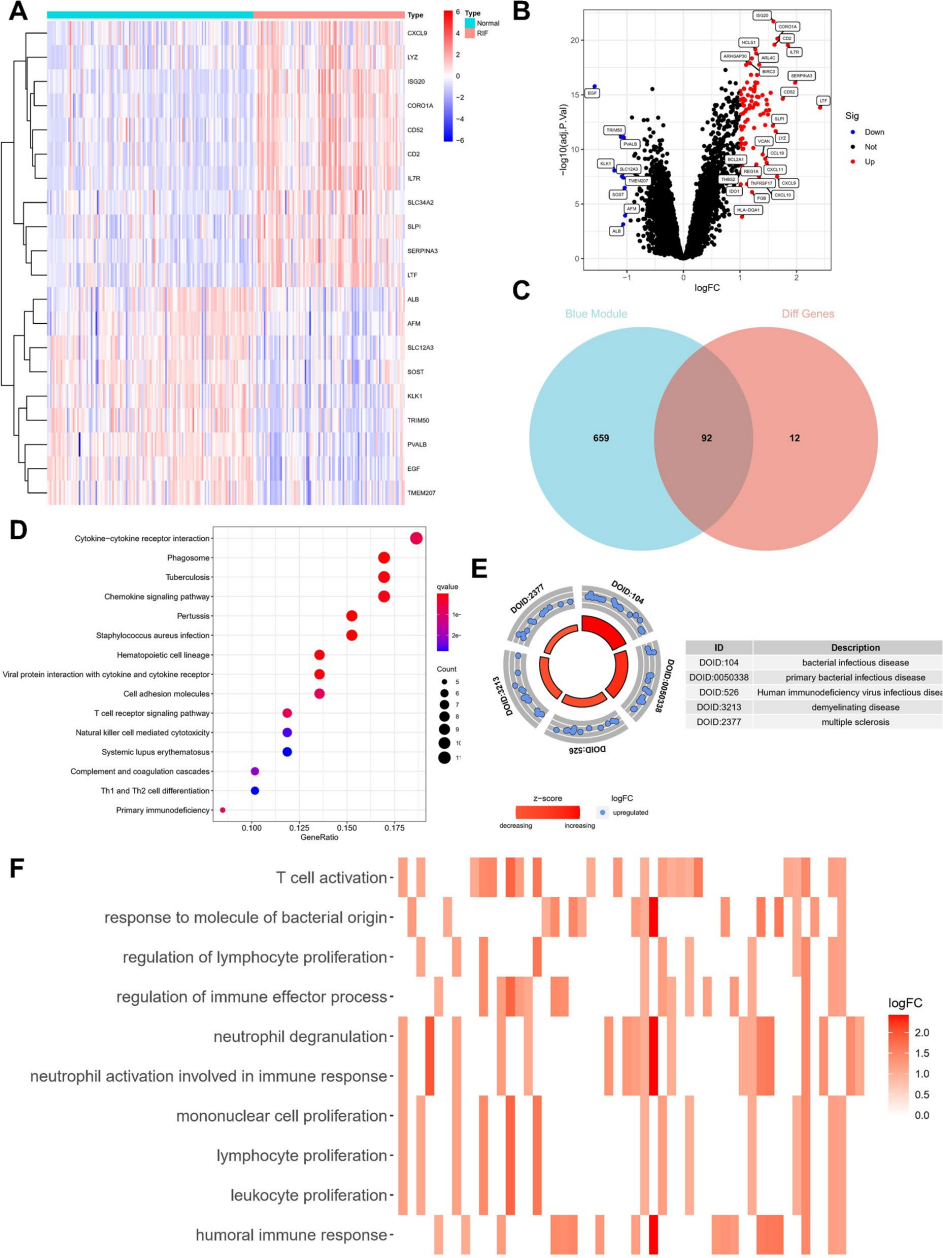
Identification of potential core genes of RIF. **A** Heat map of DEGs between normal and RIF samples. **B** Volcano map of DEGs. **C** Venn diagram of the intersection of DEGs and genes in the blue module. **D** Bubble diagram showed KEGG analysis of intersection genes (E) DO analysis of intersection genes (**F**) The heatmap showed GO analysis of intersection genes

Furthermore, the correlation between module-specific genes and clinical traits was calculated. Notably, the blue module, encompassing 751 genes, exhibited the highest positive correlation with the occurrence of kidney fibrosis (r = 0.54), thus earning its designation as the core module. Subsequent differential gene expression analysis, using the limma package, led to the identification of 104 DEGs, including 95 upregulated genes and 9 downregulated genes (Fig. 2a-b). Lastly, the genes within the blue module were overlapped with the DEGs, yielding the identification of 92 potential core genes.

### Enrichment analysis in overlap genes

To unravel the potential biological mechanisms driving the development of kidney fibrosis, KEGG analysis elucidated specific pathways, including the Chemokine signaling pathway, Cytokine-cytokine receptor interaction, and T cell receptor signaling pathway (Fig. 2d). DO analysis revealed disease categories sharing common pathogenic mechanisms, encompassing bacterial infectious disease, primary bacterial infectious disease, and Human Immunodeficiency Virus infectious disease (Fig. 2e). Further GO analysis unveiled significant enrichment of processes such as T cell activation, humoral immune response, and neutrophil degranulation (Fig. 2f). These results underscore the influence of immune-related factors on the progression of kidney fibrosis. GSEA analysis, integrating the gene set with the expression matrix, highlighted the significant involvement of pathways such as ALLOGRAFT_REJECTION and ANTIGEN_PROCESSING_AND_PRESENTATION (Additional file 1: Figure S1). In summary, a robust evidence chain suggests that immune pathways and pathways related to bacterial infection may regulate kidney fibrosis.

### Exploration of hub biomarkers

**Fig. 3:**
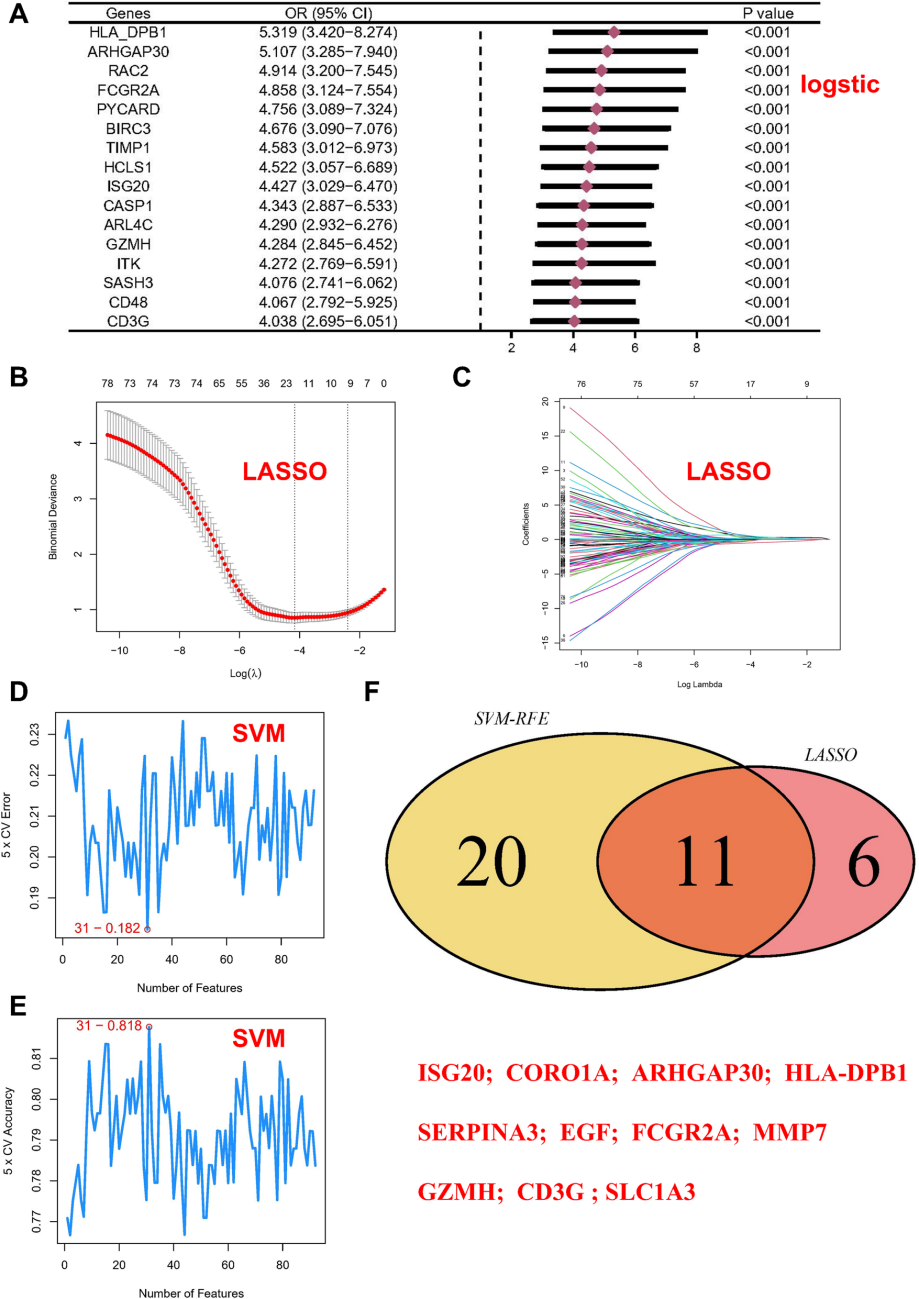
Machine learning in screening candidate diagnostic biomarkers for RIF pathogenesis.** A** forest plot of genes with an OR greater than 4. **B** Plot of LASSO partial likelihood deviance. **C** Plot of LASSO coefficient profiles. Accuracy (**D**) and error (**E**) of fivefold cross-validation (CV) in SVM-RFE algorithms, respectively.**F** Venn diagram showing the characteristic genes shared by LASSO and SVM-RFE algorithms

Logistic analysis was performed to determine the odds ratios (OR) of each overlapping gene, clarifying their contributions to kidney fibrosis development. The majority of potential core genes exhibited a risk effect, with a forest plot displaying genes having an OR greater than 4 (Fig. 3a). LASSO regression with tenfold cross-validation was then applied to further eliminate redundant genes, resulting in the selection of 17 potential genes (Fig. 3b, c). Within the overlapping genes, an SVM machine learning method was employed for further screening, revealing that when 31 genes were included, the root mean square error (RMSE) was minimized (Fig. 3d, e). Finally, the genes identified by the above algorithms were overlapped, leading to the identification of ISG20, CORO1A, ARHGAP30, HLA-DPB1, SERPINA3, EGF, FCGR2A, MMP7, GZMH, CD3G, and SLC1A3 as potential core genes (Fig. 3f).

**Fig. 4:**
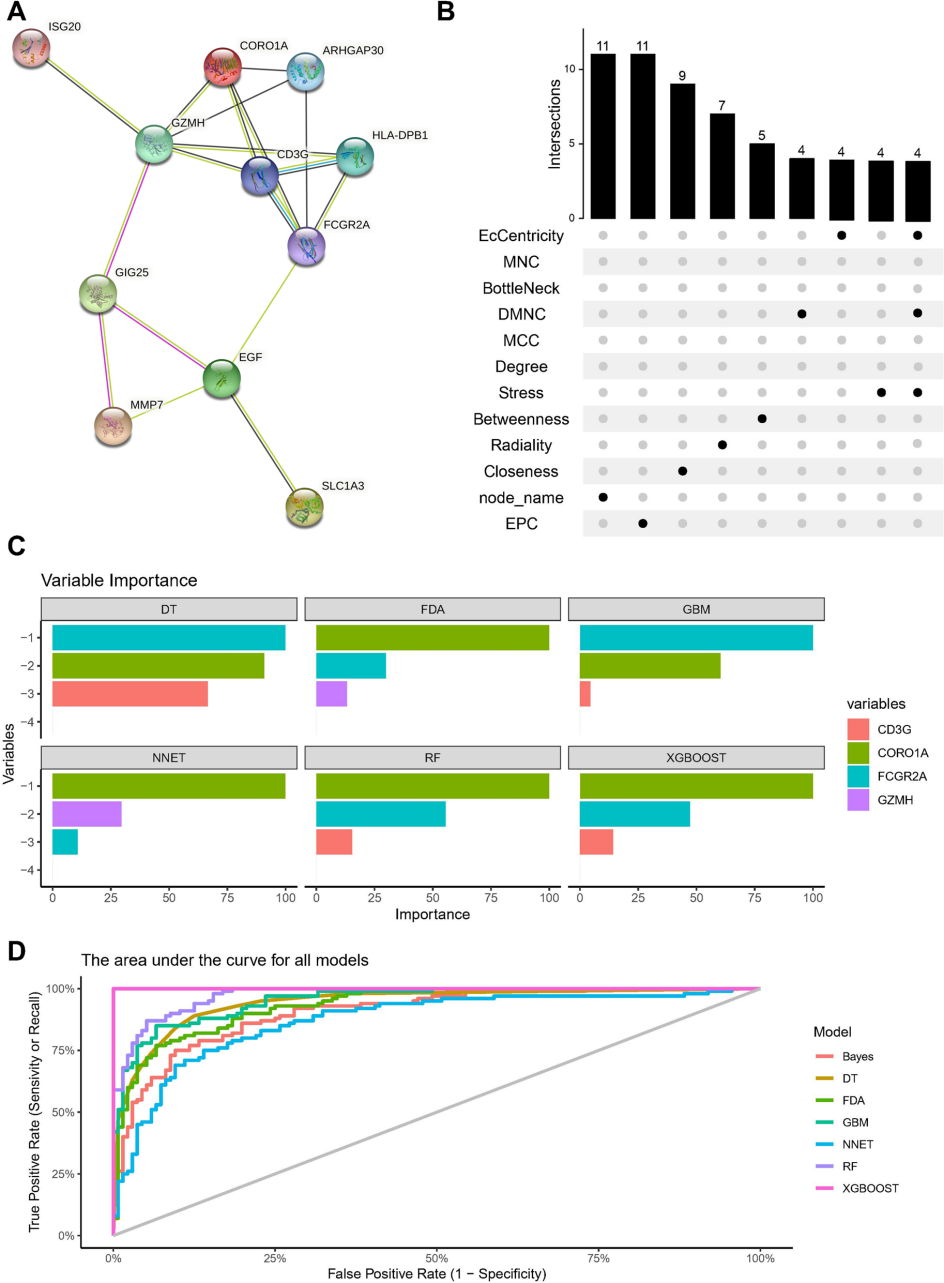
Multiple algorithms calculate the topological rank of each gene to determine the core gene based on the maximum overlap. **A** Protein–protein interaction (PPI) networks. **B** Multiple algorithms are used in Cytoscape to calculate the topological rank of each gene. **C** Various machine learning methods were employed, including Bayes, Decision Tree (DT), Fisher Discriminant Analysis (FDA), Gradient Boosting Machine (GBM), Neural Networks (NNET), Random Forest (RG), and XGBoost. **D** The area under curve (AUC) for all the above mentioned models

Subsequently, these genes were input into the STRING database to construct a protein–protein interaction (PPI) network (Fig. 4a). Various algorithms in Cytoscape were utilized to calculate the topological features of each gene, and the maximum overlap of different methods was employed to determine the core genes (CD3G, CORO1A, FCGR2A, GZMH) (Fig. 4b). Analysis of single-cell data from kidney fibrosis further confirmed the expression of the identified core genes (Additional file 1: Figure S3).

### Validation of hub biomakers

To assess the diagnostic performance of the combination of the four core genes (CD3G, CORO1A, FCGR2A, GZMH) for kidney fibrosis, various machine learning methods, including Bayes, Decision Tree (DT), Fisher Discriminant Analysis (FDA), Gradient Boosting Machine (GBM), Neural Networks (NNET), Random Forest (RG), and XGBoost, were employed. In most machine learning algorithms, CORO1A and FCGR2A consistently ranked first in importance (Fig. 4c). Notably, the XGBoost model demonstrated the highest AUC (Fig. 4d).

**Fig. 5:**
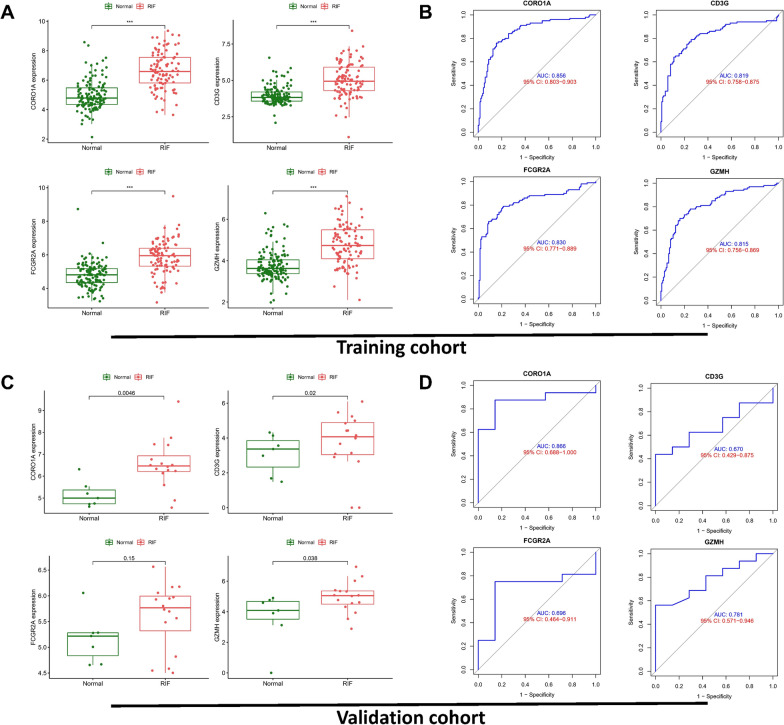
The genes have good predictive performance in the training cohort and validation cohort.** A** Differential expression analysis of the four core genes (CD3G, CORO1A, FCGR2A, GZMH) in the training cohort. **B** ROC analysis of the four core genes (CD3G, CORO1A, FCGR2A, GZMH) in the training cohort. External validation was performed on the GSE65326 dataset, and the differential expression analysis of the four core genes (**C**) and ROC analysis of the four core genes (**D**) were shown respectively

Subsequent ROC analysis and differential expression analysis on the two genes in the screening set revealed that all core genes were significantly upregulated in kidney fibrosis samples (Fig. 5a) and exhibited good predictive performance in the screening set: CORO1A (AUC = 0.856), CD3G (AUC = 0.819), FCGR2A (AUC = 0.830), GZMH (AUC = 0.815) (Fig. 5b). External validation using the GSE65326 dataset showcased similar expression patterns for most core genes in kidney fibrosis tissues, demonstrating upregulated expression (Fig. 5c). Furthermore, they exhibited robust diagnostic performance: CORO1A (AUC = 0.866), CD3G (AUC = 0.670), FCGR2A (AUC = 0.696), GZMH (AUC = 0.781) (Fig. 5d). The findings were further confirmed in an additional external validation set: GSE53605, consisting of 10 RIF samples and 45 non-RIF samples. The validation results show a high level of consistency with the training set (Additional file 1: Figure S5).

Additionally, the regulatory networks of FCGR2A and CD3G were visualized, and TF-mRNA-miRNA networks were constructed. Potential candidate compounds targeting these genes were predicted with the aim of improving symptoms in patients with kidney fibrosis (Additional file 1: Figure S2). However, it is regrettable that CORO1A and GZMH were not involved in the construction of the network.

### Analysis of differences in immune microenvironment

**Fig. 6:**
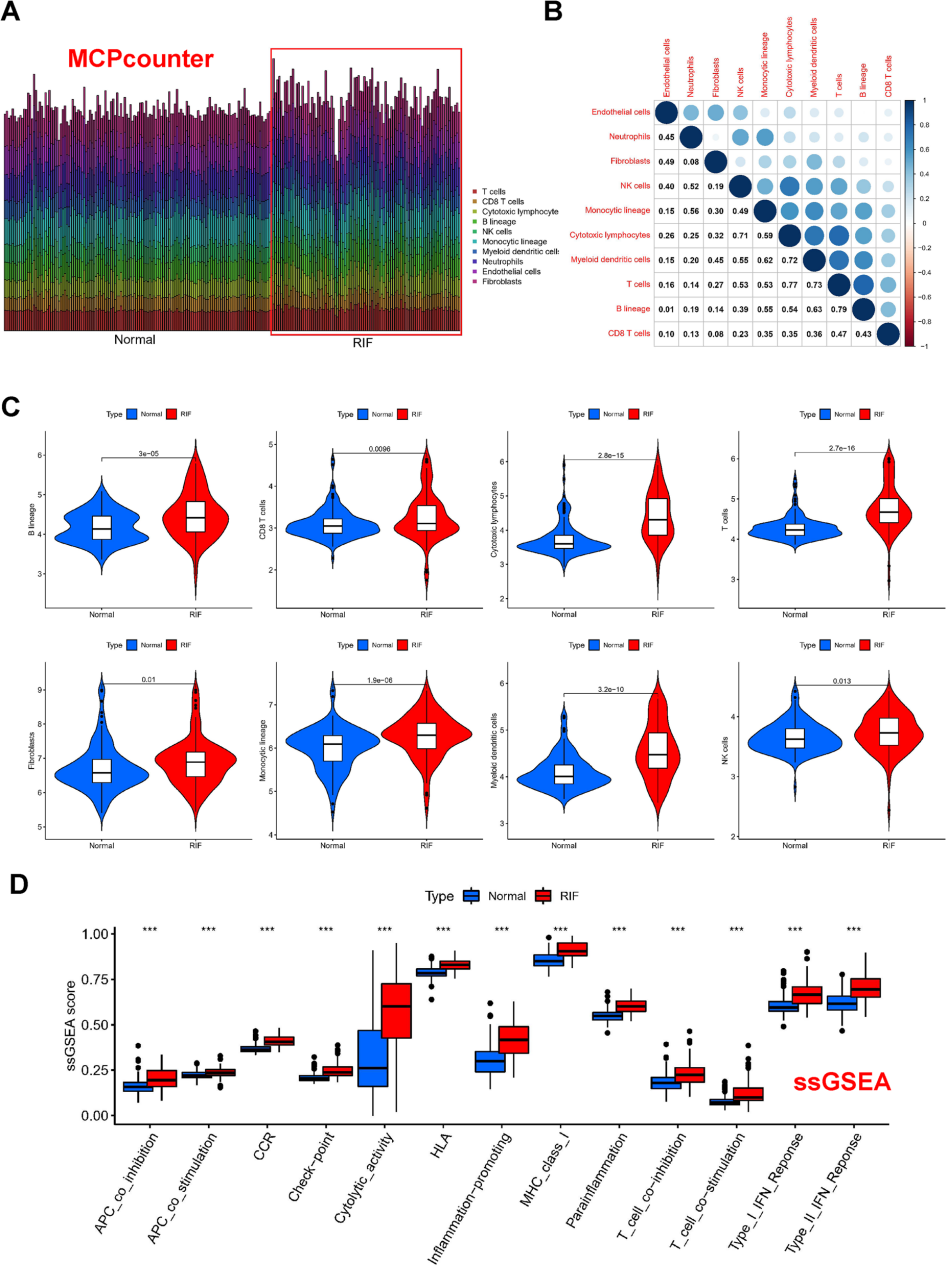
Analysis of differences in immune microenvironment.** A** Using the MCP counter, the bar plot provides an overview of the distribution of immune cells. **B** Heatmap of the correlation between immune cell types. **C** Violin plots of B lineage, CD8 T cells, Cytotoxic lymphocytes, Endothelial cells, Fibroblasts, Monocytic lineage, Myeloid dendritic cells, Neutrophils, NK cells, and T cells in normal and kidney fibrosis samples. **D** ssGSE analysis of pathways in normal and kidney fibrosis samples

Considering the significant role of immune-related pathways in kidney fibrosis observed in gene enrichment analysis (Fig. 2), we analyzed the immune cell composition in different samples using the MCPcounter algorithm. The bar plot provides an overview of the distribution of immune cells (Fig. 6a), while the heatmap demonstrates the correlation between immune cell types in detail (Fig. 6b). In kidney fibrosis tissues, B lineage and T cells showed the strongest positive correlation (r = 0.79). Results from the Wilcoxon test revealed higher levels of B lineage, CD8 T cells, Cytotoxic lymphocytes, Endothelial cells, Fibroblasts, Monocytic lineage, Myeloid dendritic cells, Neutrophils, NK cells, and T cells in kidney fibrosis samples (Fig. 6c). Moreover, most immune pathways were significantly activated in kidney fibrosis tissues (Fig. 6d).

**Fig. 7:**
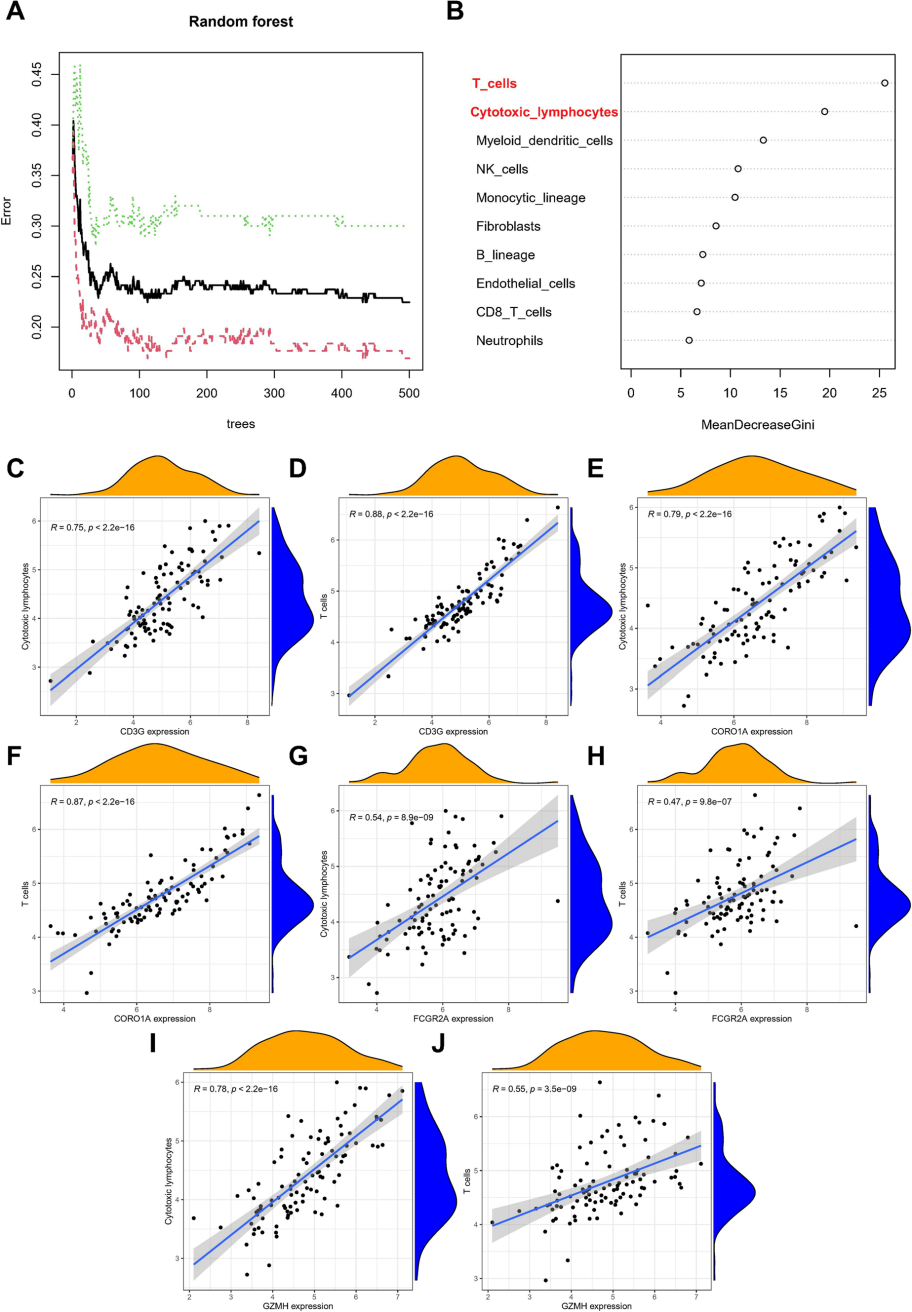
Correlation analysis of immune cells and hub biomarkers. **A** Illustration of minimal depth of a variable in the random forest tree analysis. (**B**) The different immune cell types in random forest tree analysis **C**–**J** correlation analysis between the core immune cells and the four core biomarkers

Furthermore, to identify the core immune cells that may alter the immune microenvironment in renal tissue, a random forest tree analysis was performed on immune cells (Fig. 7a, b), leading to the identification of two core immune cells that may influence the development of kidney fibrosis: Cytotoxic lymphocytes and T cells.

To further explore our core genes expression in the core immune cells, we then performed more detailed single-cell data analysis, with the dataset sourced from GSE183837. Single-cell analysis using singleR annotation in this dataset revealed 8 different cell types (Additional file 1: Figure S6A, B), and our core genes showed significantly higher expression in monocytes and NK cells (Additional file 1: Figure S6C). In cell communication analysis, it was found that after RIF, secretion signals such as KIT and VEGF were significantly upregulated (Additional file 1: Figure S6D). We also conducted pseudo-temporal analysis on the critical cell subtypes in this dataset (monocytes, NK cells), and the results indicated that in RIF samples, monocytes may undergo 6 different cell developmental fates, with two nodes. The two genes primarily expressed in monocytes, CORO1A, showed the lowest expression in fate1 and the highest in fate6. Similarly, FCGR2A exhibited the lowest expression in fate6 and the highest in fate2 (Additional file 1: Figure S6E-H). This suggests that these two genes may have opposing functions in monocyte differentiation and development.

### Correlation analysis of immune cells and hub biomarkers

In kidney fibrosis tissues, correlation analysis was conducted between the core immune cells and the four core biomarkers (Fig. 7c–j). The results revealed a positive correlation between all core genes and core immune cells, implying that these core genes may play a role in promoting immune system activation in kidney fibrosis.

### Experimental validation of elevated expression of hub biomarkers in a murine model of renal fibrosis

**Fig. 8:**
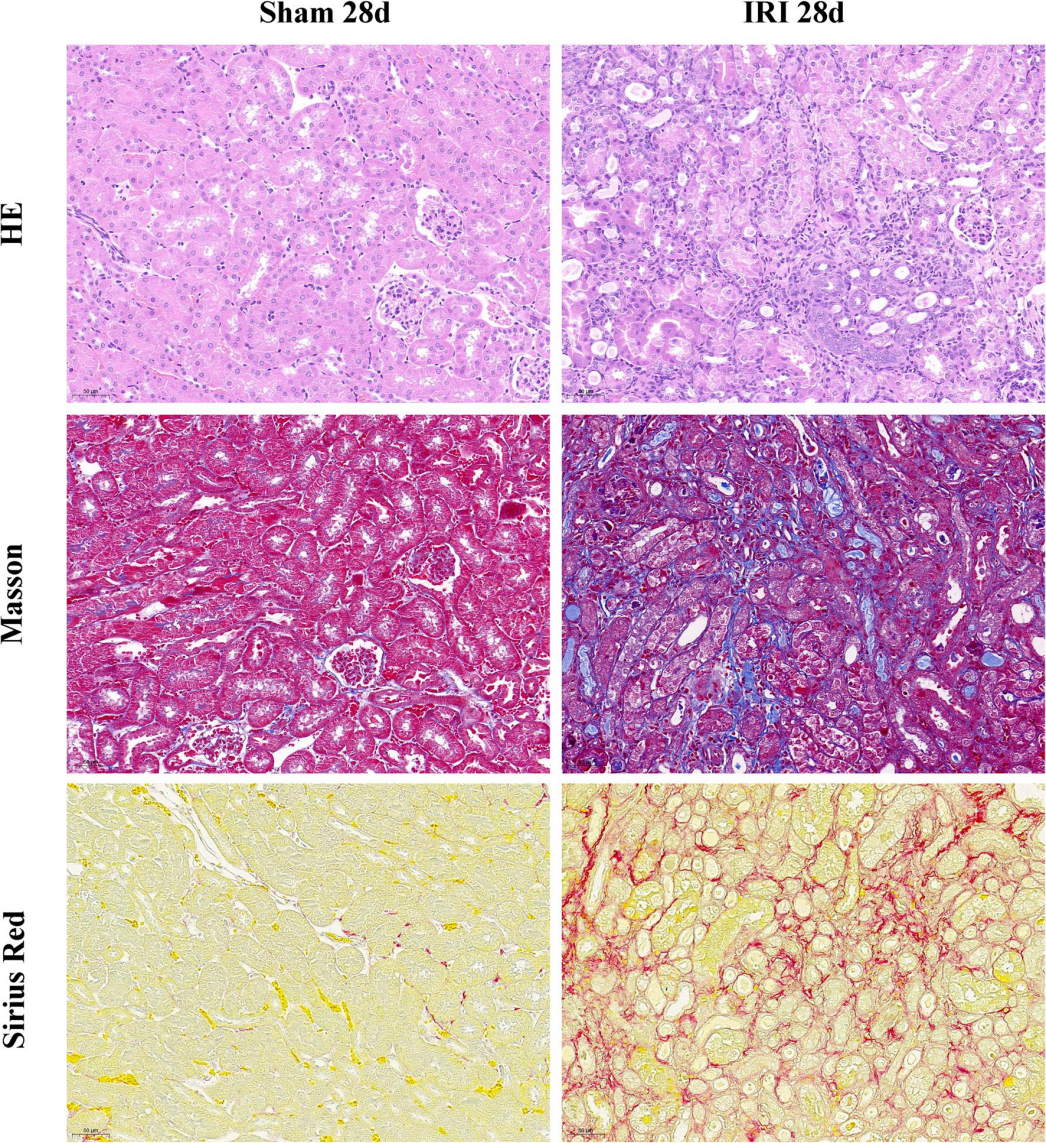
Histological analysis of the renal tissues of murine model. On day 28 after the operation, tubular injury was measured by H&E,while renal fibrosis was measured by Sirius Red and Masson trichrome staining. Scale bar: 50 μm

**Fig. 9:**
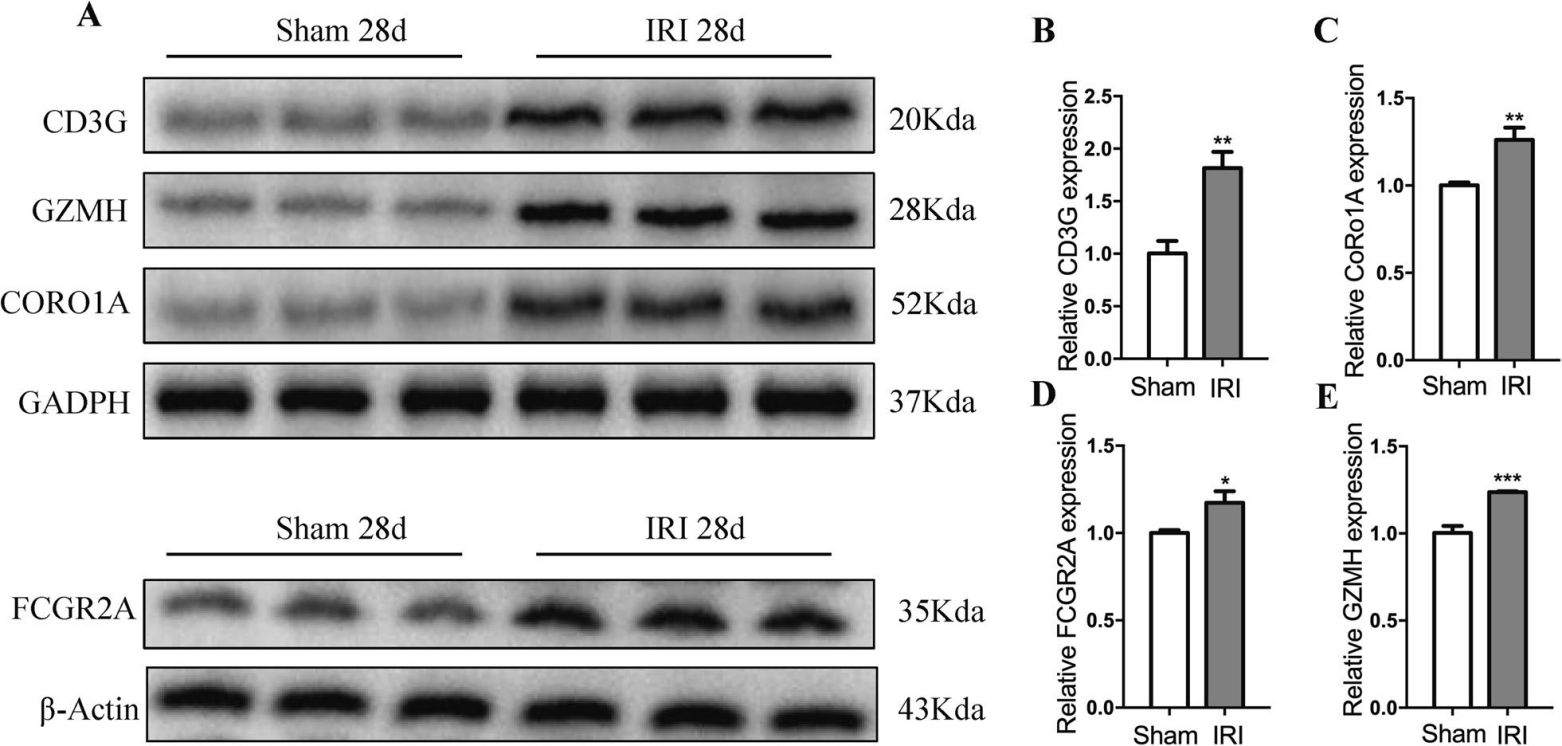
Experimental validation of elevated expression of hub biomarkers. **A** The protein expression levels of CD3G, GZMH, CORO1A and FCGR2A in the mice kidney from sham group and IRI group were detected by western blotting. The mRNA expression of CD3G (**B**), CORO1A (**C**), FCGR2A (**D**) and GZMH (**E**) in from sham group and IRI group was detected by qPCR and normalized to GAPDH expression Values were expressed as mean ± standard deviation (mean ± SD). *p < 0.05; **p < 0.01; ***p < 0.001.

To further validate the increased expression of hub biomarkers in renal fibrotic tissue, we established a mice model of renal fibrosis induced by unilateral ischemia–reperfusion injury (IRI). H&E staining revealed the presence of inflammation and fibrotic phenotypes in the kidneys of mice in the IRI group, while Sirius red and Masson staining also showed that the IRI significantly induced renal fibrosis in the mice model (Fig. 8). Under Sirius red staining, collagen fibers display a distinct red color. In the IRI model, a complex network of collagen deposition can be observed in the renal tubular interstitium, glomeruli, and around blood vessels. This network primarily consists of Type I and III collagen, indicating the regions where fibrosis occurs. In Masson's trichrome staining, collagen-rich areas appear deep blue, providing a comprehensive visualization of the fibrotic regions in the IRI model. In contrast, the control group does not exhibit such deep blue fibrotic areas. This staining method effectively highlights and delineates the fibrotic areas induced by IRI in the kidneys, showcasing the presence of collagen deposits (Fig. 8). While Western blot (Fig. 9a) and qPCR (Fig. 9 b-e) of the above mentioned samples confirmed elevated protein and mRNA levels of the four hub biomarkers in the renal fibrotic tissues of mice compared to the control group. Moreover, immunohistochemistry indicated that the positive expression of CD3G, CORO1A, FCGR2A, and GZMH was significantly increased in the IRI group after 28 days (Additional file 1: Figure S7). Our experiments corroborate the findings from the bioinformatics analysis.

### Screening for potentially effective molecules targeting the hub biomarkers through molecular docking and molecular dynamics simulations

To explore potentially effective molecules targeting the four biomarkers, we initiated a search for protein structures from UniProt using CD3G, CORO1A, FCGR2A, and GZMH as keywords. We filtered for human species in the database and selected CD3G_HUMAN (UniProt ID: P09693), COR1A_HUMAN (UniProt ID: P31146), FCG2A_HUMAN (UniProt ID: P12318), GRAH_HUMAN (UniProt ID: P20718) full-length AlphaFold predicted structures as protein structure files. Simultaneously, we downloaded a total of 2115 small molecule structure files of FDA-approved drugs from the ZINC database, performing a total of 42115 virtual screenings. For each docking simulation, we predicted 10 binding conformations, and the best binding score was utilized for sorting and selection. Duplicates were removed, and the top 20 drug small molecules with strong binding effects were retained for molecular docking. A total of 420 molecular dockings were conducted, and the heat maps of the optimal binding scores for molecular docking of the four proteins and small molecules are displayed in Additional file 1: Figure S4.

**Fig. 10:**
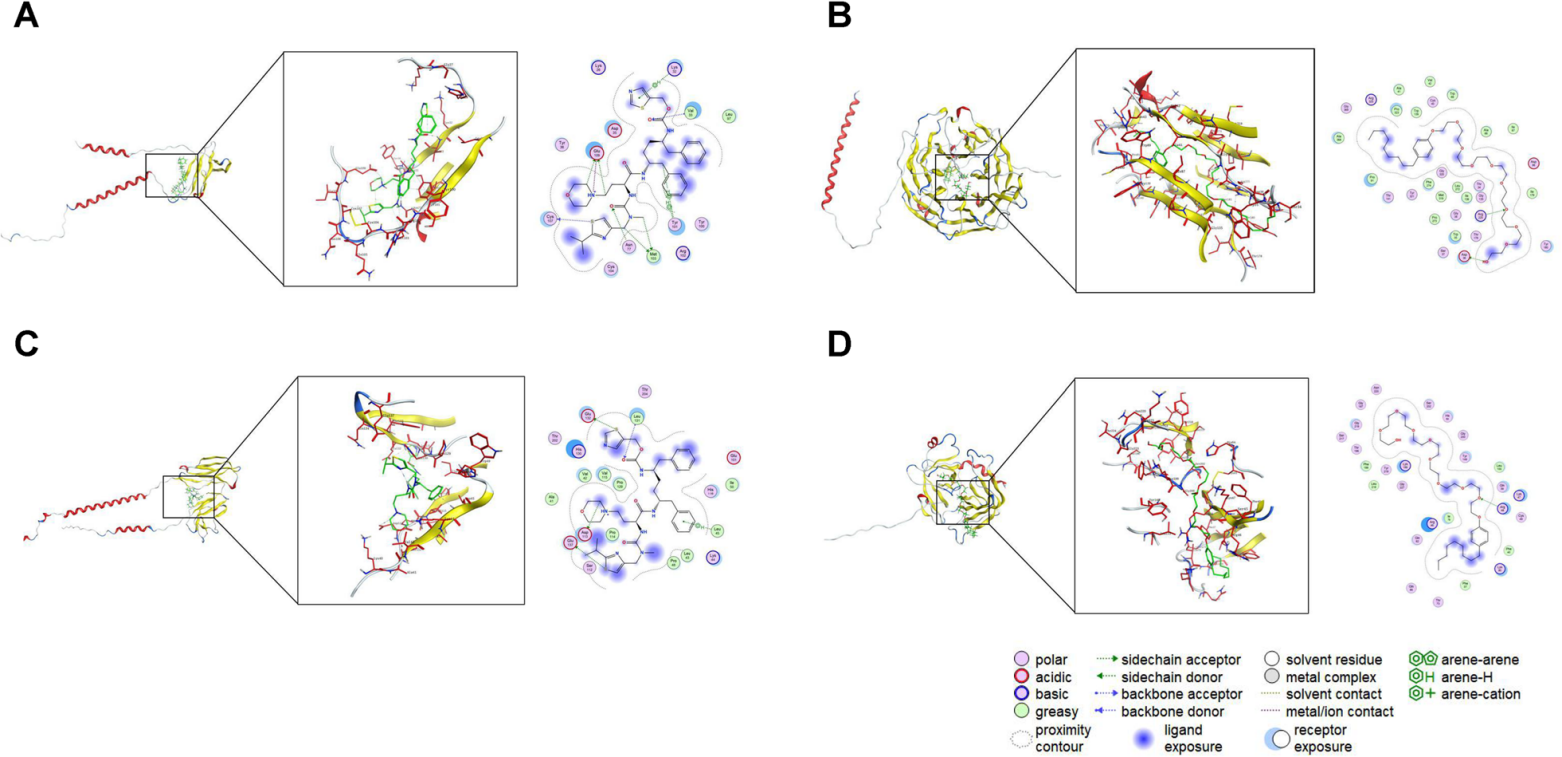
Molecular docking of the four hub biomarkers and drug small molecules.** A** Site1 of protein CD3G can dock to drug ZINC000085537014 (Cobicistat). There are hydrogen bonding interactions between the drug molecule and amino acid residues LYS-32, VAL-33, ASN-77, TYR-101, MET-103, CYS-107, GLU-109 of protein CD3G. Docking score: − 8.91646957. **B** Site1 of protein CORO1A can dock to drug ZINC000008214629 (Nonoxynol-9) with hydrogen bonding interactions between the drug molecule and amino acid residues ASP-36, ARG-225 of protein CORO1A. Docking score: − 12.3709917. **C** Site3 of protein FCGR2A can dock to drug ZINC000085537014 (Cobicistat) with hydrogen bonding interactions between the drug molecule and amino acid residues LEU-45, ASP-113, LEU-131, GLU-132, GLU-137 of protein FCGR2A. Docking score: − 9.60169411. **D** Site1 of protein GZMH can dock to drug ZINC000008214629 (Nonoxynol-9) with hydrogen bonding interaction between drug molecule and amino acid residue ARG-48 of protein GZMH. Docking score: − 10.4395981

**Fig. 11:**
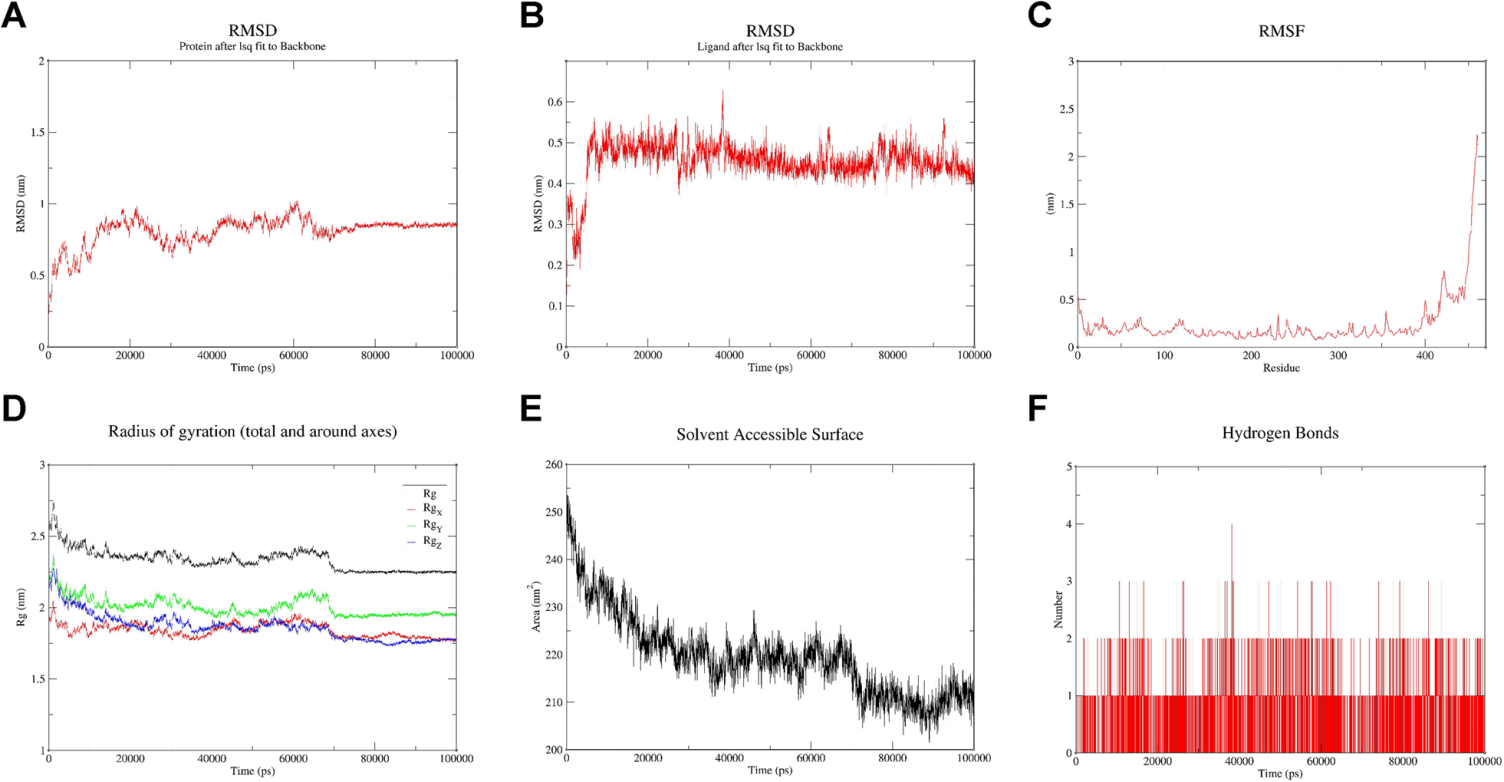
Molecular dynamics simulations of CORO1A and ZINC000008214629. **A** Between 70 ns-100 ns, the mean RMSD of the proteins in the complex was 0.845, with a variance of 0.000278 and a standard deviation of 0.016668. **B** Between 70 ns and100 ns, the mean RMSD of the ligand in the complex was 0.452, with a variance of 0.000775 and a standard deviation of 0.027835. And as can be seen by RMSF (**C**), Rg (**D**), SASA (**E**) and Hbond (**F**), after 70 ns the system enters a steady state, in which the protein wraps ligand tightly and a certain number of hydrogen bonding interactions are present

Among the 4*20 molecular docking results, four molecules—ZINC000003830276 (Tessalon), ZINC000003944422 (Norvir), ZINC000008214629 (Nonoxynol-9), and ZINC000085537014 (Cobicistat)—demonstrated the ability to bind to all four proteins. We selected the best-binding drug small molecule with each protein for interaction analysis (Fig. 10). The most satisfactory binding molecule and protein (CORO1A and ZINC000008214629) were then chosen for dynamic simulation to further validate the reliability of the molecular docking results. The results, including RMSD (Root Mean Square Deviation), RMSF (Root Mean Square Fluctuation), Rg (Radius of Gyration), Hydrogen bond, and SASA (Solvent Accessible Surface Area) of the protein, indicated that after 70 ns, the protein progressively compacted, and the solvent-accessible area decreased significantly. These findings suggested that when the system reached a steady state, the protein tightly enveloped the drug small molecule, with the presence of a certain number of hydrogen bonding interaction forces (Fig. 11).

## Discussion

Two methods are utilized to assess post-kidney transplant rejection reactions, including invasive renal biopsy and non-invasive biomarkers. Non-invasive biomarkers encompass markers detected in blood or urine, such as the enzyme-linked immunosorbent assay (ELISA) for collagen protein fragments [25], or the chemiluminescent microparticle immunoassay for serum human epididymis secretory protein 4 (HE4) [26]. While these biomarkers offer the convenience of non-invasive diagnostic, their accuracy is limited. Leveraging invasive kidney tissue samples, we conducted biomarker discovery through RNA sequencing, which provides higher throughput and accuracy. Moreover, this approach is closely linked to the pathogenic mechanisms, facilitating the development of novel therapeutic targets.

Interstitial fibrosis is an independent risk factor that significantly influences transplant kidney outcomes and, consequently, clinical decisions. Assessing the degree of fibrosis in the transplanted kidney contributes to evaluating treatment efficacy. Several diagnostic biomarkers have been identified in previous studies, throwing light on the value of biomarkers in the early detection of potential kidney allograft failure [27, 28]. While in this study, drawing from transcriptome and single-cell sequencing data of patients with kidney fibrosis in public databases, we identified four core genes significantly associated with renal fibrosis, namely CD3G, CORO1A, FCGR2A, and GZMH. Multiple machine learning methods were employed to verify the diagnostic effectiveness of these four core genes in combination, demonstrating their strong predictive performance in the validation dataset. This research provides novel insights for the future biological diagnosis and future treatment of post-kidney transplant fibrosis.

The four hub biomarkers, namely CD3G, CORO1A, FCGR2A, and GZMH, are intricately linked to post-transplant rejection. CD3G encodes the CD3-gamma peptide, a vital component of the T-cell receptor-CD3 complex, which cooperates with CD3-epsilon, -delta, and -zeta, as well as T-cell receptors α/β and γ/δ. This complex plays a pivotal role in linking antigen recognition to various intracellular signal transduction pathways. Mutations in this gene are associated with T-cell immunodeficiency [29]. CD3G closely correlates with post-transplant acute rejection [30], a significant risk factor for kidney fibrosis [31, 32]. CORO1A, encoded by the CORO1A gene, is a member of the WD repeat protein family. WD repeat sequences, typically flanked by glycine-histidine and tryptophan-aspartate (GH-WD) motifs, are about 40 amino acids in length and may facilitate the formation of heterotrimeric or multiprotein complexes. Members of this family partake in various cellular processes, including cell cycle progression and signal transduction [33]. CORO1A serves as a critical biological marker for renal interstitial fibrosis (RIF) and is associated with the degree of immune infiltration in RIF [34]. FCGR2A, another hub biomarker, belongs to the immunoglobulin Fc receptor gene family and is expressed on the surface of various immune response cells. It is primarily found on phagocytic cells like macrophages and neutrophils, playing a pivotal role in the phagocytosis and clearance of immune complexes [35]. Polymorphisms within this gene are linked to susceptibility to conditions such as chronic periaortitis, characterized by fibrosis [36]. The GZMH gene encodes a member of the serine proteinase peptidase S1 family. This protein is constitutively expressed in NK (natural killer) cells of the immune system and may participate in cytotoxicity during innate immune responses, inducing target cell death and directly cleaving substrates within pathogen-infected cells [37]. NK cells have also been reported to have functional relevance in fibrotic diseases [38].

Sequencing analyses of past samples from post-kidney transplant fibrosis have indicated that complex interactions between immune and renal cells contribute to transplant renal fibrosis [39], and there is heterogeneity in immune responses among patients [40]. Our research findings support these discoveries, revealing elevated levels of B cell lineage, CD8 T cells, cytotoxic lymphocytes, endothelial cells, fibroblasts, monocyte lineage, myeloid dendritic cells, neutrophils, NK cells, and T cells in kidney fibrosis samples. This reflects the complexity of cell types involved in the immune response post-kidney transplant. Most immune pathways were significantly activated in kidney fibrosis tissues, and a random forest tree analysis on immune cells led to the identification of two core immune cells that may influence the development of kidney fibrosis: Cytotoxic lymphocytes and T cells. These findings align with previous research conclusions, as Cytotoxic lymphocytes and T cells are essential for allograft rejection following kidney transplantation, with acute rejection being a major risk factor for post-transplant kidney fibrosis [32]. Simultaneously, the expression of the four hub biomarkers, including CD3G, CORO1A, FCGR2A, and GZMH, correlates positively with the extent of infiltration by core immune cells in kidney fibrosis. These results suggest that the high expression of these hub biomarkers not only serves a diagnostic role but may also be involved in regulating the immune response that promotes fibrosis.

While previous studies have identified biomarkers through RNA sequencing of kidney fibrosis tissue samples and blood samples or protein detection [41, 42], our work holds significance and innovation. Firstly, the biomarkers obtained through the analysis of RNA sequencing samples from multiple sources were not only validated in the validation set but also confirmed in the expression trends observed in single-cell sequencing samples. More importantly, based on molecular docking analysis of biomarker proteins, we identified four potential drugs for the treatment of kidney fibrosis, providing crucial clues for future clinical interventions.

However, our study has certain limitations. Firstly, like many other studies we chose the murine IRI induced renal fibrosis model for the validation of the hub biomarkers [24, 43]. However, this might not be the best model to accurately reflect the pathophysiologic process of post-transplant renal fibrosis. In future studies, the inclusion of human post-transplant renal biopsy samples or the application of murine kidney transplant models could help to reach more solid conclusions. Furthermore, the diagnostic model composed of the four hub biomarkers requires validation through large-scale clinical trials involving more patients. Moreover, further research is needed to elucidate the specific mechanisms through which these four hub biomarkers participate in the immune response and immune cell activation, which could open doors to new therapeutic targets for kidney fibrosis.

In conclusion, employing logistic analysis, tenfold cross-validation with LASSO, and gene topological analysis in Cytoscape, we identified CD3G, CORO1A, FCGR2A, and GZMH as significantly associated with kidney fibrosis. Machine learning methods further validated the robust predictive performance of these four genes in the validation dataset. Experimental verification through Western blotting (WB) and quantitative PCR (qPCR) confirmed the elevated expression of these core genes in a mouse model of kidney fibrosis. The positive correlation observed between all core genes and core immune cells suggests that these genes may play a role in amplifying the activity of the immune system in kidney fibrosis.

Additionally, through molecular docking and molecular dynamics simulations, we comprehensively pinpointed four potentially effective small molecules: ZINC000003830276 (Tessalon), ZINC000003944422 (Norvir), ZINC000008214629 (Nonoxynol-9), and ZINC000085537014 (Cobicistat). These findings offer valuable insights for the future clinical biological diagnosis and treatment of kidney fibrosis.

### Supplementary Information

**Additional file 1: Figure S1.** GSEA analysis of gene sets with expression matrices. **Figure S2.** TF-mRNA-miRNA networks of FCGR2A and CD3G. **Figure S3.** Analysis of single-cell data from kidney fibrosis. **Figure S4.** Heat maps of the top20 binding scores for each molecular docking of four proteins and small molecules. **Figure S5.** The genes have good predictive performance in the additional external validation cohort. **Figure S6.** Single-cell data analysis with singleR annotation, cell communication analysis and pseudo-temporal analysis. **Figure S7.** Immunohistochemistry analysis of the renal tissues of the murine model.

**Additional file 2.** **Table S1.** The detailed PCR primer sequences.

## Data Availability

The datasets during and/or analysed during the current study available from the corresponding author on reasonable request.
