# Supplementary figures and images for: Characterizing hub biomarkers for post-transplant renal fibrosis and unveiling their immunological functions through RNA sequencing and advanced machine learning techniques
Source: J Transl Med. 2024 Feb 20;22:186. doi: 10.1186/s12967-024-04971-9 (PMC10880303; doi:10.1186/s12967-024-04971-9)

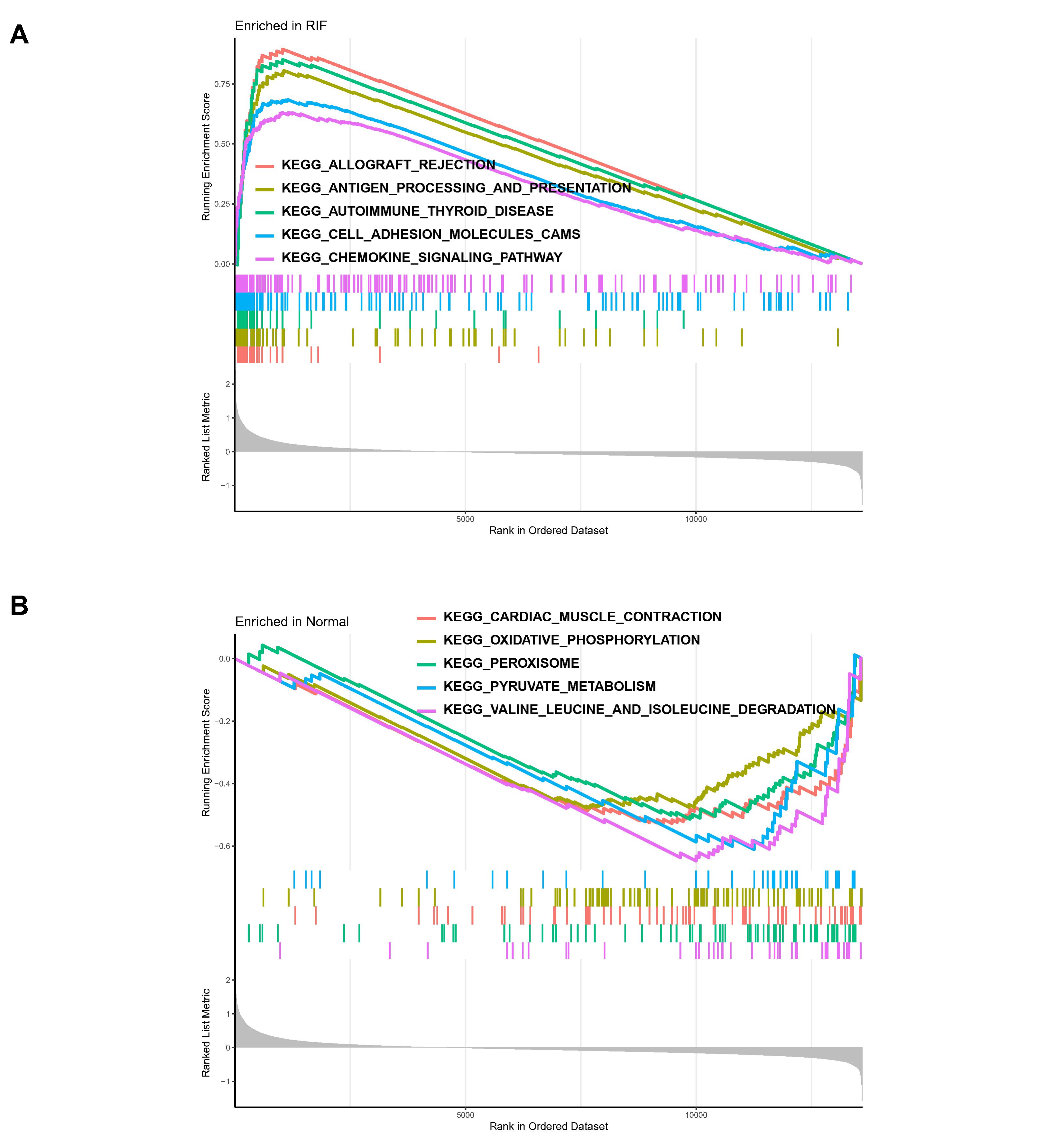

Supplement: Supplementary file 1 — Additional file 1: Figure S1. GSEA analysis of gene sets with expression matrices. Figure S2. TF-mRNA-miRNA networks of FCGR2A and CD3G. Figure S3. Analysis of single-cell data from kidney fibrosis. Figure S4. Heat maps of the top20 binding scores for each molecular docking of four proteins and small molecules. Figure S5. The genes have good predictive performance in the additional external validation cohort. Figure S6. Single-cell data analysis with singleR annotation, cell communication analysis and pseudo-temporal analysis. Figure S7. Immunohistochemistry analysis of the renal tissues of the murine model. [file 12967_2024_4971_MOESM1_ESM.zip › supple figs/FigureS1.tiff]

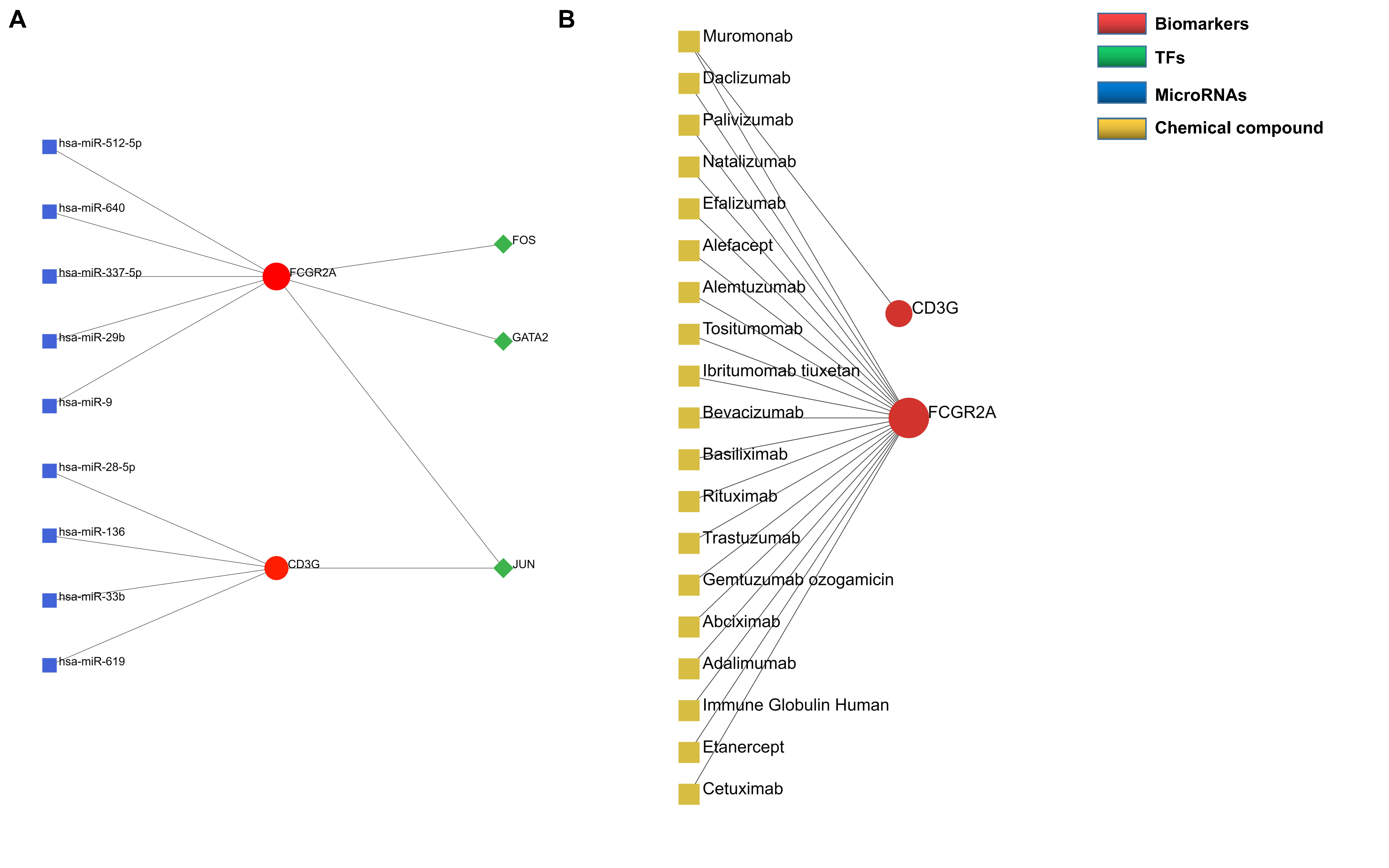

Supplement: Supplementary file 1 — Additional file 1: Figure S1. GSEA analysis of gene sets with expression matrices. Figure S2. TF-mRNA-miRNA networks of FCGR2A and CD3G. Figure S3. Analysis of single-cell data from kidney fibrosis. Figure S4. Heat maps of the top20 binding scores for each molecular docking of four proteins and small molecules. Figure S5. The genes have good predictive performance in the additional external validation cohort. Figure S6. Single-cell data analysis with singleR annotation, cell communication analysis and pseudo-temporal analysis. Figure S7. Immunohistochemistry analysis of the renal tissues of the murine model. [file 12967_2024_4971_MOESM1_ESM.zip › supple figs/figureS2.tiff]

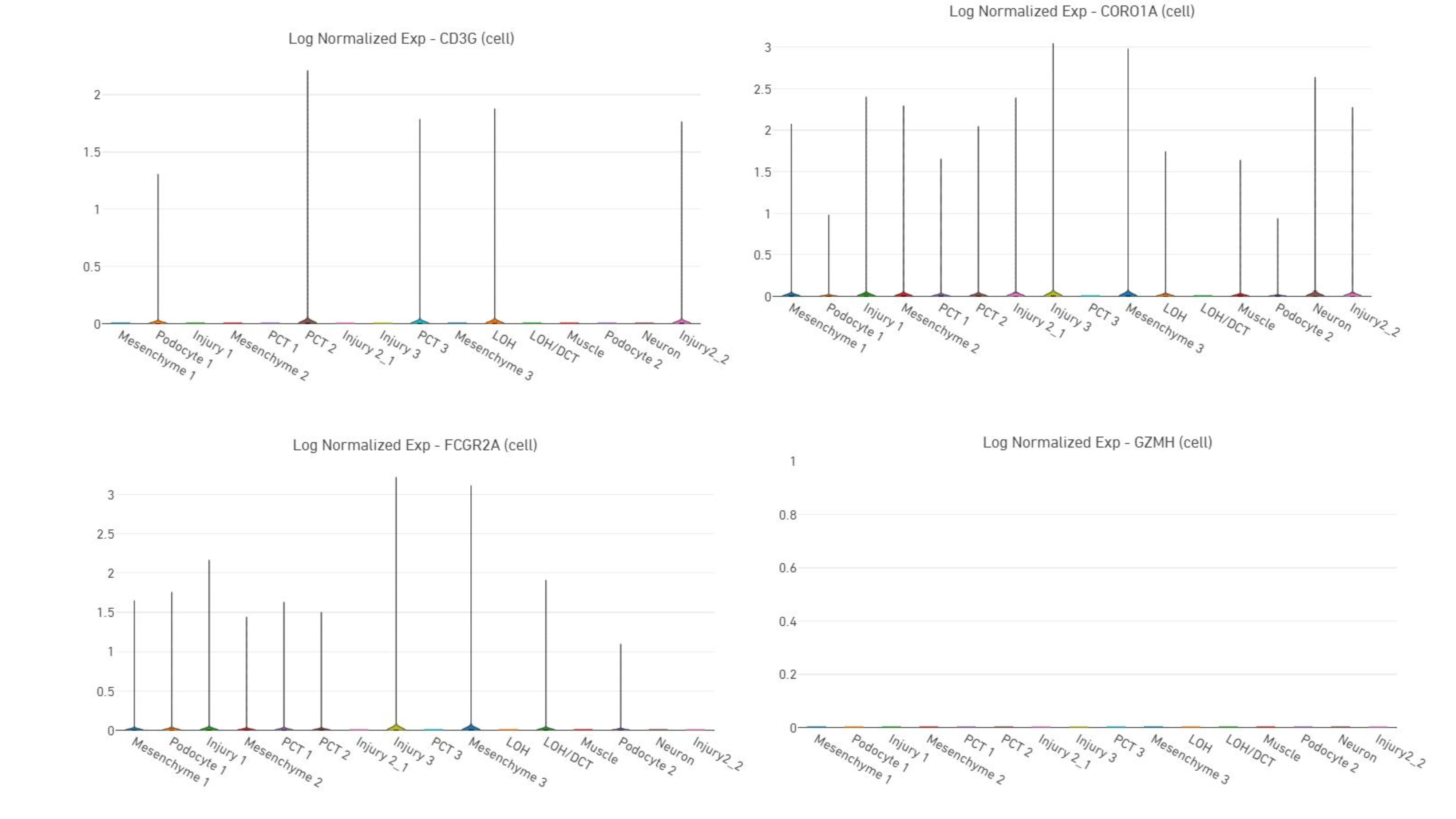

Supplement: Supplementary file 1 — Additional file 1: Figure S1. GSEA analysis of gene sets with expression matrices. Figure S2. TF-mRNA-miRNA networks of FCGR2A and CD3G. Figure S3. Analysis of single-cell data from kidney fibrosis. Figure S4. Heat maps of the top20 binding scores for each molecular docking of four proteins and small molecules. Figure S5. The genes have good predictive performance in the additional external validation cohort. Figure S6. Single-cell data analysis with singleR annotation, cell communication analysis and pseudo-temporal analysis. Figure S7. Immunohistochemistry analysis of the renal tissues of the murine model. [file 12967_2024_4971_MOESM1_ESM.zip › supple figs/FigureS3.tiff]

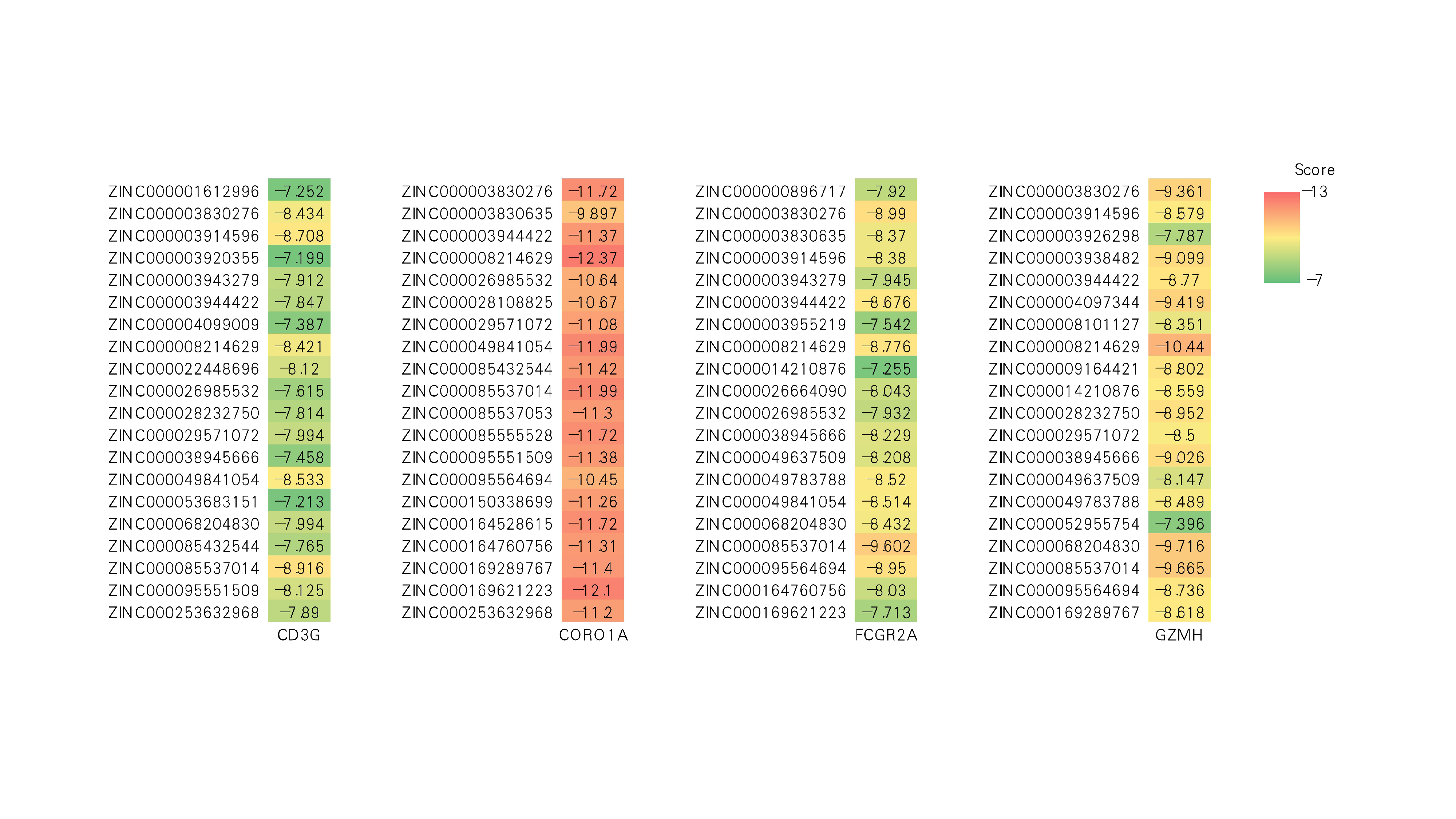

Supplement: Supplementary file 1 — Additional file 1: Figure S1. GSEA analysis of gene sets with expression matrices. Figure S2. TF-mRNA-miRNA networks of FCGR2A and CD3G. Figure S3. Analysis of single-cell data from kidney fibrosis. Figure S4. Heat maps of the top20 binding scores for each molecular docking of four proteins and small molecules. Figure S5. The genes have good predictive performance in the additional external validation cohort. Figure S6. Single-cell data analysis with singleR annotation, cell communication analysis and pseudo-temporal analysis. Figure S7. Immunohistochemistry analysis of the renal tissues of the murine model. [file 12967_2024_4971_MOESM1_ESM.zip › supple figs/FigureS4.tiff]

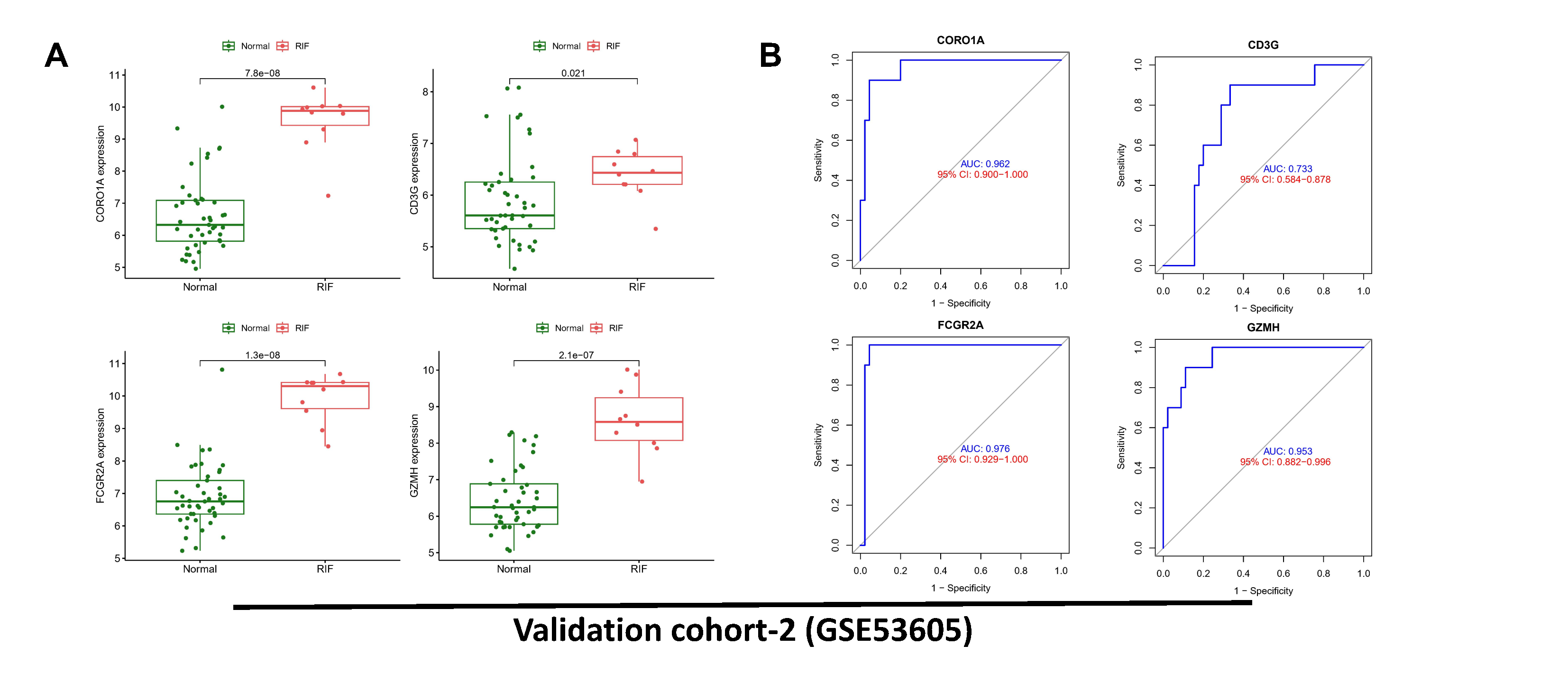

Supplement: Supplementary file 1 — Additional file 1: Figure S1. GSEA analysis of gene sets with expression matrices. Figure S2. TF-mRNA-miRNA networks of FCGR2A and CD3G. Figure S3. Analysis of single-cell data from kidney fibrosis. Figure S4. Heat maps of the top20 binding scores for each molecular docking of four proteins and small molecules. Figure S5. The genes have good predictive performance in the additional external validation cohort. Figure S6. Single-cell data analysis with singleR annotation, cell communication analysis and pseudo-temporal analysis. Figure S7. Immunohistochemistry analysis of the renal tissues of the murine model. [file 12967_2024_4971_MOESM1_ESM.zip › supple figs/FigureS5.png]

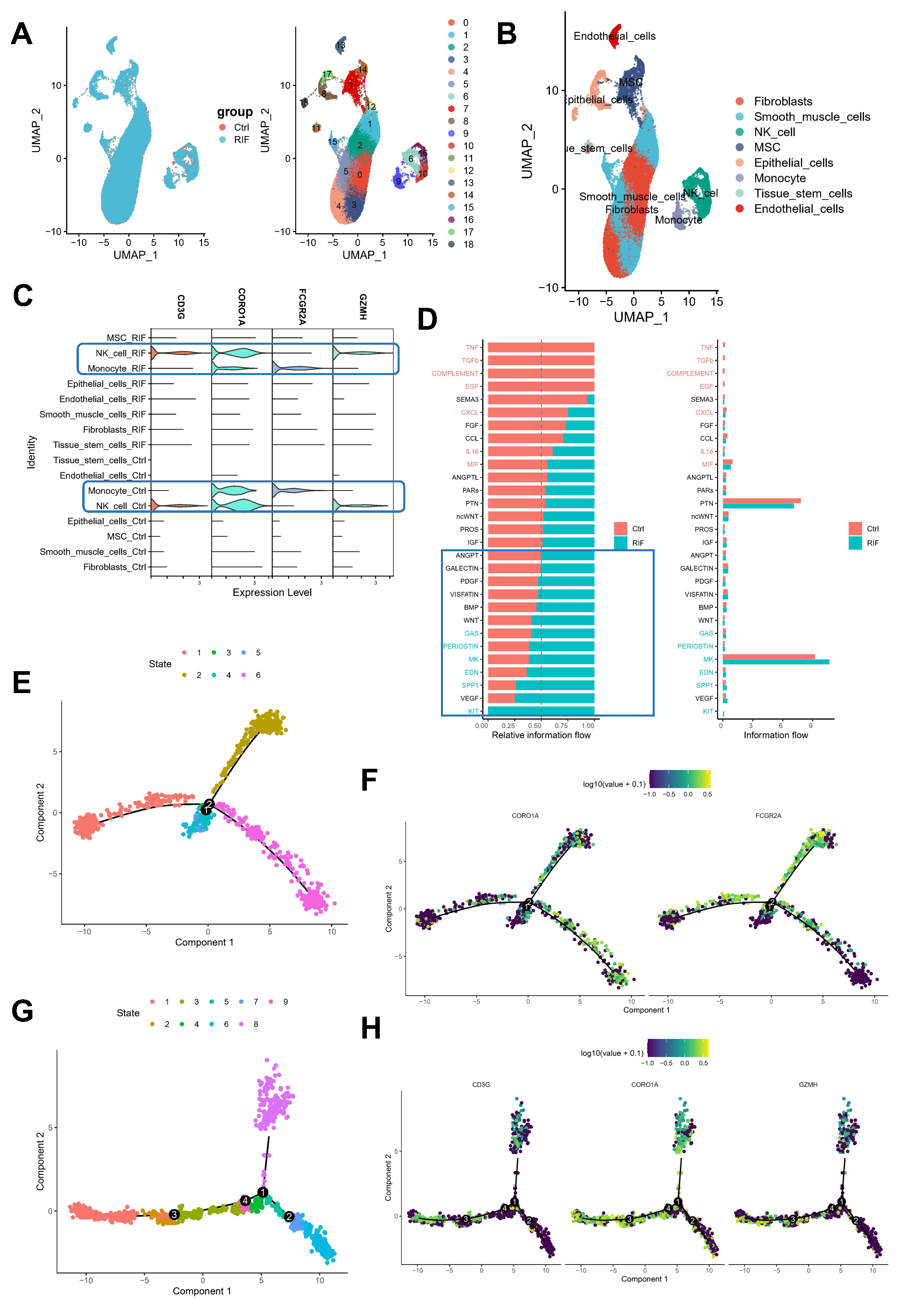

Supplement: Supplementary file 1 — Additional file 1: Figure S1. GSEA analysis of gene sets with expression matrices. Figure S2. TF-mRNA-miRNA networks of FCGR2A and CD3G. Figure S3. Analysis of single-cell data from kidney fibrosis. Figure S4. Heat maps of the top20 binding scores for each molecular docking of four proteins and small molecules. Figure S5. The genes have good predictive performance in the additional external validation cohort. Figure S6. Single-cell data analysis with singleR annotation, cell communication analysis and pseudo-temporal analysis. Figure S7. Immunohistochemistry analysis of the renal tissues of the murine model. [file 12967_2024_4971_MOESM1_ESM.zip › supple figs/FigureS6.tif]
