# Supplementary material for: Characterizing hub biomarkers for post-transplant renal fibrosis and unveiling their immunological functions through RNA sequencing and advanced machine learning techniques
Source: J Transl Med. 2024 Feb 20;22:186. doi: 10.1186/s12967-024-04971-9 (PMC10880303; doi:10.1186/s12967-024-04971-9)
Supplement: Supplementary file 2 — Additional file 2. Table S1. The detailed PCR primer sequences. [file 12967_2024_4971_MOESM2_ESM.docx]

| Gene Name | Forward Primer(5'-3') | Reverse Primer(5'-3') |
| --- | --- | --- |
| CORO1A | GGCAGAGCAGATGGGATGAG | CCATACCACGCTGAGACTCC |
| CD3G | ACATCAAACCCCCTGCAAGT | AGTCTGCTTGTCTGAAGCTCT |
| FCGR2A | GTACTATCTGCCAAGCCGGG | AGCAGCAGCCAGTCAGAAAT |
| GZMB(GZMH) | CTGCTCACTGTGAAGGAAGTATAA | TCAGCTCTAGGGACGATGGG |
